# Evaluation of Telemental Health Utilization and Patient Engagement at King Abdulaziz Medical City: A Retrospective Cross-Sectional Study of the Pre-Pandemic, Pandemic, and Post-Pandemic Periods (2019–2025)

**DOI:** 10.7759/cureus.113791

**Published:** 2026-08-01

**Authors:** Thamer M Aledreesi, Abdulmajeed A Abukhamis

**Affiliations:** 1 Health Informatics, King Saud bin Abdulaziz University for Health Sciences, Riyadh, SAU; 2 Health Informatics, King Abdulaziz Medical City, Riyadh, SAU; 3 College of Public Health &amp; Health Informatics, King Saud bin Abdulaziz University for Health Sciences, Riyadh, SAU

**Keywords:** covid-19, electronic health records, health informatics, patient engagement, saudi arabia, telemental health, telepsychiatry

## Abstract

Introduction

The use of health services expanded during the pandemic and became a regular part of healthcare. There is not a lot of information about how these services are used over the long term and how patients engage with them. This study examined how patients used telemental health services at King Abdulaziz Medical City (KAMC), Riyadh, Kingdom of Saudi Arabia, from 2019 to 2025.

Objectives

To evaluate how telemental health services were used and how patients engaged with them during the time before the pandemic, during the pandemic, and after the pandemic. The study also compared how many appointments were completed and the characteristics of patients who had virtual appointments versus in-person appointments.

Methods

This was a retrospective, repeated cross-sectional census study of electronic health record data on all scheduled mental health appointments, virtual and in-person, at KAMC from January 1, 2019, to December 31, 2025. All eligible encounters and unique patients across 12 facilities were included using total enumeration rather than sampling. Data were cleaned and analyzed in Python using Google Colab. Descriptive statistics were used to summarize appointment and patient characteristics. Chi-square tests were used to compare categorical variables, including appointment completion by encounter type, and the Mann-Whitney U test was used to compare patient age between virtual and in-person appointments. A binary logistic regression model was used to identify factors associated with appointment completion, including encounter type, pandemic period, and patient demographics.

Results

The study included 479,643 appointments with 51,323 patients at 12 facilities. Overall, 9% of the appointments were virtual. In 2019, there were no appointments, but their use increased during the pandemic and then stayed steady at around 11% to 12% per year after 2021. Overall, 55% of appointments were completed. Virtual appointments had a higher completion rate than in-person appointments, with 60% of virtual appointments being completed compared to 54% of in-person appointments. We also found that patients who had virtual appointments were substantially older and that virtual appointments were mostly used for follow-up visits rather than new patient appointments.

Conclusion

Telemental health services went from being an emergency solution during the pandemic to a part of mental health services at the facility. The study found that virtual appointments were associated with patient engagement, which means that patients were more likely to complete their appointments. These findings support the continued use of a hybrid of in-person and virtual mental health services. The findings provide information to support the planning and development of telemental health services. Telemental health services are a part of the healthcare system, and they can be an effective way to deliver the services.

## Introduction

Telemental health expansion in Saudi Arabia

Telemental health services are scaling across the Kingdom of Saudi Arabia and around the world [1-4], especially during the COVID-19 pandemic, when most healthcare services switched to being provided virtually. This transition of care has been sustained as a core component of the national digital health transformation; for example, the Seha Virtual Hospital, which is a flagship digital health initiative of Saudi Arabia's Ministry of Health, has expanded specialized telehealth consultation in their services [1,2]. Virtual healthcare is considered a good clinical fit because it relies primarily on verbal and visual communication. The service delivery can be achieved via video conferences and other remote modalities [5].

Global telehealth expansion during COVID-19

During the acute COVID-19 period (early 2020), health systems worldwide shifted rapidly to virtual care. Notably, telehealth encounters rose sharply as social distancing to prevent the spread of infection was introduced to enable remote assessment and treatment [3,4]. In mental health services, this rapid conversion maintained the continuity of care [6]. Additionally, surveys showed high patient acceptance and a substantial willingness to continue virtual visits after the acute phase [6]. Several institution-level series and cohort studies reported lower appointment no-show rates for telehealth compared with in-person visits during the pandemic. This supported telemental health as an effective substitution when face-to-face access was restricted [7-9]. These pandemic-era operational changes created stress-tested digital clinical workflows [7-9]. Moreover, a method of measurement approach justifies the seven-year institutional monitoring [7-9].

Transition from pandemic to post-pandemic telemental health

Telemental health operational patterns have shifted when comparing the acute pandemic phase to the current post-pandemic era [10,11]. During the COVID-19 crisis, virtual care functioned primarily as an emergency measure for infection control [3,4]. This became a substitute for many in-person visits, leading to marked reductions in no-show rates that were partly driven by lack of physical alternatives [8,9]. In the post-pandemic period, the landscape has evolved toward a sustainable (hybrid) model, with virtual volumes stabilizing below pandemic peaks but above pre-2020 levels. In addition, patient preference for convenience is becoming a principal driver of virtual uptake [10,11]. In Saudi Arabia, the temporary digital channels established during the outbreak have been formalized into permanent national platforms and integrated into broader health transformation plans [1,2,12].

The Saudi Arabian experience with telemental health

Recent indexed studies in Saudi Arabia report high patient satisfaction with telemental health while also documenting operational limitations. These include inconsistent specialty coding, variable survey response rates, heterogeneous appointment workflows, and intermittent technical interruptions during virtual consultations [13-15]. National analyses and reviews of Arabic-language mental health applications further document large user uptake of culturally adapted digital services. This illustrates parallel growth in app-based mental health utilization that is relevant to institutional planning [12,16,17]. Together, these Saudi studies support the clinical appropriateness, policy alignment, and practical feasibility of ongoing, multi-year institutional monitoring of telemental health services [13-15].

International evidence and emerging trends

Global systematic reviews and meta-analyses confirm that telemental health achieves outcomes and diagnostic concordance comparable to face-to-face care. This was for many conditions, and patients commonly report high satisfaction. However, these reviews highlight heterogeneity in measurement instruments and a relative paucity of longitudinal hospital-level descriptive series reporting standardized workforce and utilization indicators (for example, encounters per active provider) [10,18,19]. Emerging international evidence describes operational trends toward hybrid care models (initial in-person visit followed by virtual follow-up). In addition, the experimental integration of artificial-intelligence tools (for example, sentiment analysis) augments clinician assessment in telemental health [19,20].

Research gap and study rationale

Despite policy-level investment and encouraging local and international evidence, there is limited published, standardized, multi-year hospital-level reporting that describes service capacity, workforce participation, utilization volumes, specialty distribution, patient demographics, and patient satisfaction for telemental health in Saudi tertiary centers. Generating a seven-year institutional evidence base for King Abdulaziz Medical City, Riyadh, Kingdom of Saudi Arabia, including an encounter-level no-show comparison between telehealth and in-person services, can help address an operational and policy gap. This may directly inform workforce planning and provide measurable indicators for quality improvement and routine monitoring [1,13,18].

Aims and objectives

This study aimed to evaluate the utilization of telemental health services and patient engagement at King Abdulaziz Medical City by comparing trends across the pre-pandemic, pandemic, and post-pandemic periods from 2019 to 2025. Specifically, the study sought to compare utilization patterns between telemental health and in-person mental health encounters over time and to assess patient engagement by examining differences in no-show rates between virtual and face-to-face appointments. In addition, the study explored demographic differences among patients utilizing telemental health services compared with those attending in-person visits.

## Materials and methods

Study design

This is a retrospective repeated cross-sectional descriptive census of all eligible encounters and unique beneficiaries recorded at King Abdulaziz Medical City for the years 2019-2025. The design employs encounter-level descriptive statistics and comparative analyses to examine differences between telehealth and in-person service delivery and trend analyses to evaluate temporal changes across the seven-year period. The census approach used total enumeration of records rather than sampling; a stratified random validation sample was used for data quality checks.

Sample size

A census design was applied, and all eligible encounters and unique beneficiaries in the institutional records for January 1, 2019, through December 31, 2025, were included. No formal sample size calculation was required for enumeration.

Inclusion criteria

The study included all patients (adults, adolescents, and pediatrics) with a scheduled mental health appointment at King Abdulaziz Medical City between January 1, 2019, and December 31, 2025, for whom a completed encounter or scheduled appointment record exists in the system. Encounters were recorded as either telehealth (virtual) or in-person, as classified under mental health specialty clinics listed in the variable dictionary.

Exclusion criteria

Appointments cancelled by either the provider or the patient prior to the scheduled date and time were excluded. System test entries, training records, technical test appointments, and otherwise labelled non-clinical appointments were also excluded. Duplicate records (identical encounter identifiers or duplicate timestamped entries identified in the source systems) and records missing essential variables required for encounter classification or unique beneficiary identification were excluded.

Data preparation

The dataset was imported into Python in Google Colab and prepared for analysis. Initial processing included cleaning column labels, checking for missing values, identifying duplicate records, and reviewing the number of unique patients. Date fields were parsed to support time-based analysis, and derived fields were created for calendar year, month, and broader time period groupings.

The dataset was checked for completeness, duplicate rows, and implausible values. Derived age values were calculated only when the relevant source dates were valid. Records with implausible age values were excluded from age-related analyses. No manual source-record validation, chart review sample, or external reconciliation process was performed within the notebook.

Missingness was assessed at the record level. No imputation was performed. For regression analysis, records with missing values in model inputs were removed before modelling. Age analyses included only records with a valid derived age.

Statistical analysis

The analysis used descriptive statistics, cross-tabulations, hypothesis testing, and regression modelling. Categorical data were summarized using counts and percentages. Continuous age data were summarized using measures of central tendency and spread. Group comparisons were assessed using the chi-square testing for categorical data and non-parametric testing for age where appropriate. A logistic regression model was used to assess factors associated with the main binary outcome. Statistical significance was set at 0.05. Confidence intervals were calculated for key proportions.

Outputs generated

The notebook produced tables and figures summarizing missingness, distributions, group comparisons, time trends, cross-tabulations, and regression results.

Software

Data processing, statistical analysis, and figure generation were performed in Python 3.10.0 using Google Colab. The following libraries were used, each serving a distinct role in the analysis pipeline: NumPy 2.0.2 was used for numerical array operations and mathematical computations [21]; pandas 2.2.2 was used for data loading, column parsing, deduplication, missing-value assessment, and tabular transformations [22,23]; Matplotlib 3.10.0 was used to generate static visualizations, including bar charts, line plots, and heatmaps [24]; Seaborn 0.13.2 was used for statistical data visualization, including annotated grouped bar plots and completion-rate heatmaps [25]; SciPy 1.16.3 was used for inferential testing, specifically chi-square tests of independence and the Mann-Whitney U test [26]; and Statsmodels 0.14.6 was used for binary logistic regression modelling and computation of McFadden’s pseudo R² [27].

Ethical considerations

Ethical approval for this study was granted by the King Abdullah International Medical Research Center on 04/03/2026 under IRB Approval No. 00000304726 and Study Number NRR26/031/2. The IRB is a registered entity with the National Committee of Bioethics (Registration No.: H-01-R-005) and operates under the Ministry of National Guard Health Affairs.

## Results

Data overview and quality

The dataset contains 479,643 appointment records from 51,323 unique patients across 12 facilities, spanning seven calendar years, with 0 missing values and 0 duplicate rows. Table 1 presents a structured overview of the dataset.

**Table 1:** Dataset overview Note: unique patient count uses EnterpriseNumber as an identifier only, not as a study variable.

Characteristic	Value
Total appointment records	479,643
Unique patients (EnterpriseNumber: identifier only)	51,323
Study period	January 1, 2019, to December 31, 2025
Calendar years covered	7
Missing values (all original columns)	0 (0)
Fully duplicated rows	0 (0)
Named facilities (FacilityName)	12
Facility types	Hospital; primary health care
Unique departments (DepartmentName)	29
Unique services (ServiceName)	199
Unique nationalities (Nationality)	53

Age at appointment

Age was derived for 479,639 records (99.99%). The mean age was 32.77 years (SD = 21.44), and the median was 30.31 years (IQR: 13.63-47.61 years). Virtual encounter patients were substantially older (median = 37.98 years; mean = 40.13; SD = 17.85) than in-person patients (median = 29.54 years; mean = 32.03; SD = 21.56). Table 2 summarizes all age statistics.

**Table 2:** Summary statistics: age at appointment date (n = 479,639 valid records) Note: Age = (AppointmentDate minus BirthDate) / 365.25 days. Four records with missing BirthDate were excluded. Ages outside 0–120 years were treated as implausible and excluded.

Statistic	Value	Note
Valid age records	479,639	99.99% of dataset
Mean (years)	32.77	SD = 21.44
Median (years)	30.31	50th percentile
Q1 (25th percentile)	13.63 years	Lower quartile
Q3 (75th percentile)	47.61 years	Upper quartile
IQR	34.0 years	Q3 minus Q1
Range	0.1–110.3 years	Maximum minus minimum
Yes (virtual): median	37.98 years	n = 44,125; mean = 40.13; SD = 17.85
No (in-person): median	29.54 years	n = 435,514; mean = 32.03; SD = 21.56

All categorical variables

Table 3 presents frequency distributions for all categorical study variables. Appointments were predominantly in-person (90.8%), and 54.9% were completed. Gender was nearly equally distributed. Saudi nationals accounted for 98.1% of appointments. Married patients were the largest marital status group at 52.6%. Follow-up visits were the most common appointment type at 48.1%. Hospital-based facilities accounted for 90.5% of appointments. The post-pandemic period contained the most appointments at 65.6%, reflecting its longer four-year duration.

**Table 3:** Descriptive statistics: all categorical study variables (n = 479,643) Note: percentages were calculated against n = 479,643. Nationality shows only the dominant group; 52 additional nationalities are present in the dataset.

Variable / Category	n	Percentage (%)
Encounter type (virtual)		
Yes (virtual)	44,126	9.2%
No (in-person)	435,517	90.8%
Appointment completion status		
Completed	263,432	54.9%
Not completed	216,211	45.1%
Gender		
Female	242,229	50.5%
Male	237,414	49.5%
Marital status		
Married	252,101	52.6%
Single	151,244	31.5%
Divorced	48,042	10.0%
Widowed	20,073	4.2%
Unknown	8,183	1.7%
Appointment type		
Follow-up visit	230,824	48.1%
New patient	129,769	27.1%
First visit	119,050	24.8%
Facility type		
Hospital	434,035	90.5%
Primary health care	45,608	9.5%
Pandemic period		
Pre-pandemic (January 2019 to February 2020)	60,375	12.6%
During pandemic (March 2020 to December 2021)	104,562	21.8%
Post-pandemic (January 2022 to December 2025)	314,706	65.6%
Nationality (top of 53 nationalities)		
Saudi Arabia	470,660	98.1%
All other nationalities (52 groups)	8,983	1.9%

Utilization trends over time

**Figure 1:**
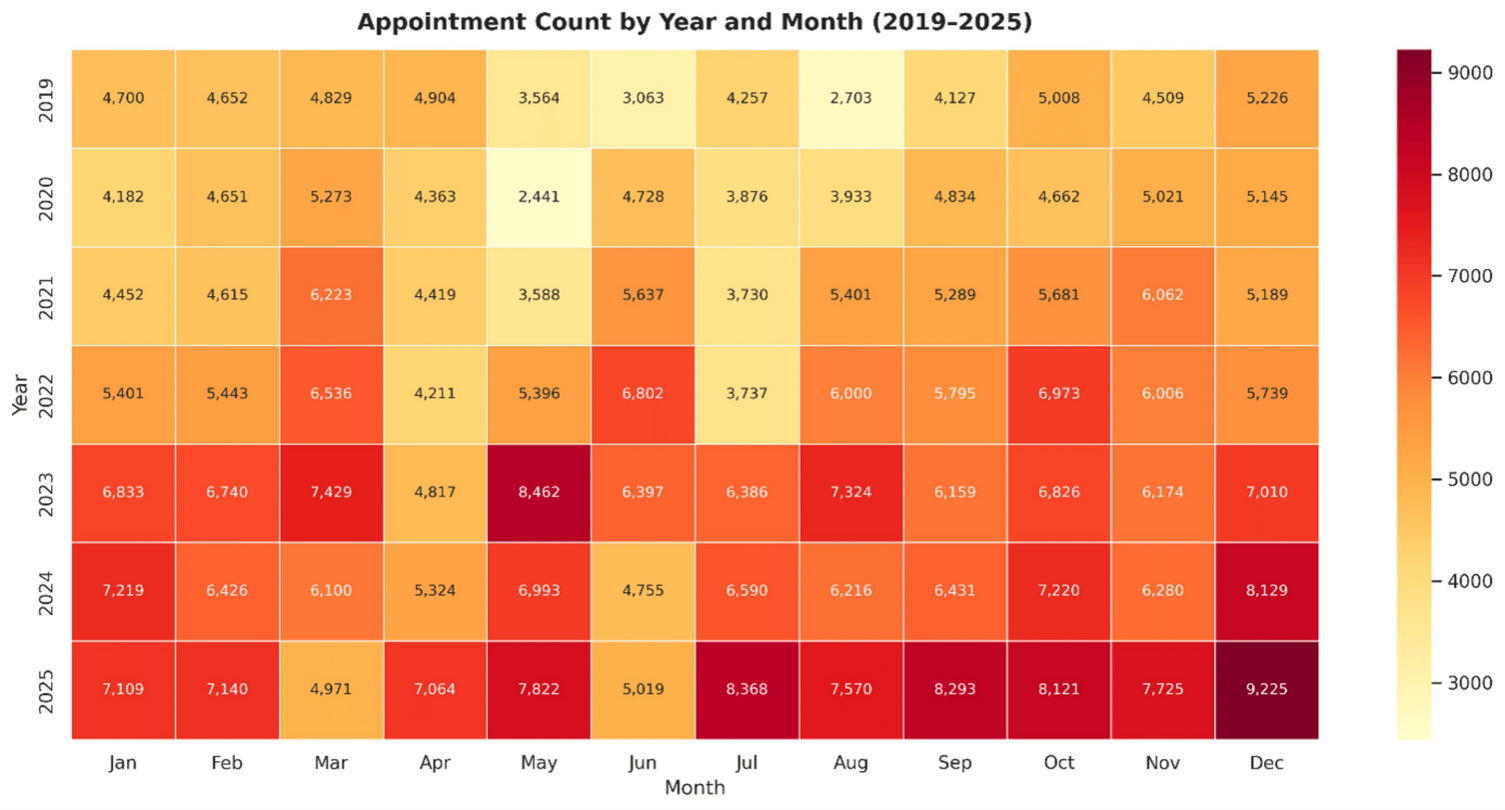
Year-by-month appointment count heatmap. Cell values show the number of appointments per month-year combination. Growing appointment volumes across years are visible, with complete data for all months.

Zero virtual appointments were recorded in 2019. Virtual encounters emerged in 2020 (n = 449; 0.8%), rose sharply in 2021 (n = 6,519; 10.8%), and stabilized at 11.2%-12.3% annually from 2022 through 2025. Total appointment volume grew steadily from 51,542 in 2019 to 88,427 in 2025 (Table 4). Figure 1 presents a year-by-month appointment count heatmap.

**Table 4:** Annual appointment volume by encounter type (2019–2025) Note: there were zero virtual encounters in 2019, as confirmed by data inspection. Completion percentages were calculated independently for each calendar year.

Year	Total	Yes (Virtual)	Virtual %	No (In-Person)	Completion %
2019	51,542	0	0.0%	51,542	50.7%
2020	53,109	449	0.8%	52,660	56.2%
2021	60,286	6,519	10.8%	53,767	55.2%
2022	68,039	7,965	11.7%	60,074	54.2%
2023	80,557	9,046	11.2%	71,511	55.0%
2024	77,683	9,232	11.9%	68,451	55.0%
2025	88,427	10,915	12.3%	77,512	54.5%
Total	479,643	44,126	9.2%	435,517	54.9%

Table 5 presents utilization by the pandemic period. The pre-pandemic period (n = 60,375) had 0 virtual appointments and a 50.9% completion rate. The pandemic period (n = 104,562) saw 6.7% virtual encounters and a 55.7% completion rate. The post-pandemic period (n = 314,706) stabilized at 11.8% virtual and 55.4% completion, confirming sustained telehealth retention alongside maintained engagement.

**Table 5:** Appointment volume, virtual utilization, and completion by the pandemic period

Pandemic Period	Total	Yes (Virtual)	Virtual %	Completed	Completion %
Pre-pandemic (January 2019 to February 2020)	60,375	0	0.0%	30,708	50.9%
During pandemic (March 2020 to December 2021)	104,562	6,968	6.7%	58,271	55.7%
Post-pandemic (January 2022 to December 2025)	314,706	37,158	11.8%	174,453	55.4%
Total	479,643	44,126	9.2%	263,432	54.9%

Patient engagement and completion analysis

**Figure 2:**
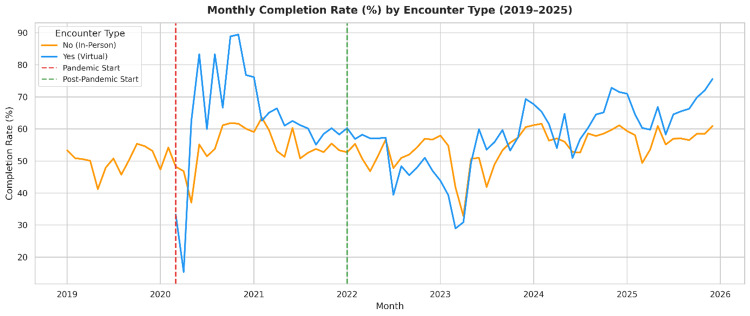
Monthly completion rate (%) by encounter type (2019–2025). Virtual encounter completion rates (blue) are consistently higher than in-person rates (orange) from 2020 onward. Both lines show month-to-month variation, but the virtual-above-in-person pattern is sustained throughout.

**Figure 3:**
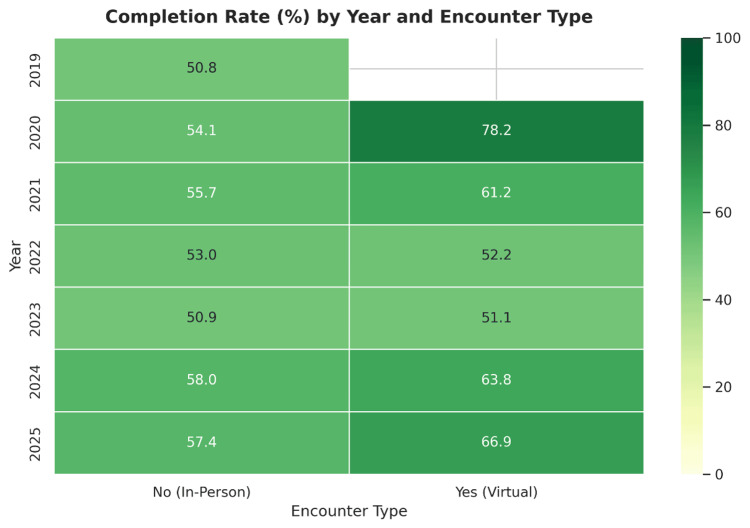
Completion rate (%) heatmap: year by encounter type. Virtual encounter completion rates are consistently higher than in-person rates from 2020 onward. The pre-pandemic row (2019) has no virtual encounters.

**Figure 4:**
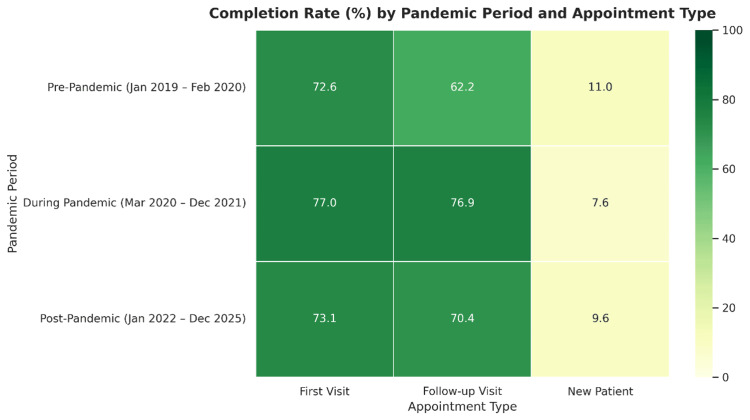
Completion rate (%) heatmap: pandemic period by appointment type. New patient completion is lowest in all periods. First visit and follow-up visit completion improved from the pre-pandemic to later periods.

The overall appointment completion rate was 54.9%. Table 6 presents completion rates with 95% Wilson score confidence intervals by encounter type. Virtual encounters had a 5.2 percentage-point higher completion rate (59.6% vs 54.4%). The confidence intervals do not overlap, confirming a statistically and observationally meaningful difference. Female patients (n = 242,229, 55.5%) had a slightly higher completion rate than male patients (n = 237,414; 54.3%). Primary health care (PHC) facilities (n = 45,608; 56.3%) showed a slightly higher completion rate than hospital-based facilities (n = 434,035; 54.7%). Follow-up visits had the highest completion rate (n = 230,824; 61.9%), followed by first visits (n = 119,050; 55.0%), and new patient appointments had the lowest completion rate (n = 129,769; 40.3%). Completion rate by marital status shows single (n = 151,244; 55.4%) to widowed (n = 20,073; 47.6%), with modest variation across groups. Completion improved from the pre-pandemic period (n = 60,375; 50.9%) to the pandemic period (n = 104,562; 55.7%) and post-pandemic period (n = 314,706; 55.4%). Figure 2 shows monthly completion trends by encounter type. Figures 3, 4 show completion rate heatmaps.

**Table 6:** Multiple linear regression predicting behavioral intention Note: 95% Wilson score CIs were computed directly from the dataset. Chi-square = 430.46, df = 1, p < 0.001, Cramer's V = 0.030. CI, confidence interval

Encounter Type	Completed (n)	Total (n)	Rate (%)	95% Wilson CI
Yes (virtual)	26,302	44,126	59.6%	59.1%–60.1%
No (in-person)	237,130	435,517	54.4%	54.3%–54.6%
Overall	263,432	479,643	54.9%	-

Virtual versus in-person encounter profile

Table 7 presents virtual encounter rates and completion rates by gender, appointment type, facility type, and marital status. Female patients had a higher virtual rate (10.5%) than male patients (7.9%). Follow-up visits and first visits had similar virtual proportions (~12%), while new patient appointments had a negligible virtual rate (0.9%) and the lowest completion rate (40.3%). Hospital facilities had a higher virtual rate (9.8%) than PHC facilities (3.1%). Married patients had the highest virtual rate (12.7%) among marital status groups. Age distribution by encounter type shows the median age: yes (virtual) = 37.98 years; no (in-person) = 29.54 years. The Mann-Whitney U test confirmed a statistically significant age difference (p < 0.001, rbc [rank-biserial correlation] = -0.194). Follow-up-visit patients were older on average than first visit or new patient appointment patients. Female and male patients showed comparable age distributions. Hospital patients were older on average than PHC patients. Pandemic and post-pandemic patients are older on average than pre-pandemic patients, partly reflecting the older age profile of virtual encounter patients introduced in 2020.

**Table 7:** Virtual encounter rate and completion rate by patient and appointment characteristics

Group	Total (n)	Yes (Virtual) (n)	Virtual %	Completion %
Gender				
Female	242,229	25,369	10.5%	55.5%
Male	237,414	18,757	7.9%	54.3%
Appointment type				
Follow-up visit	230,824	28,219	12.2%	61.9%
First visit	119,050	14,726	12.4%	55.0%
New patient	129,769	1,181	0.9%	40.3%
Facility type				
Hospital	434,035	42,701	9.8%	54.7%
Primary health care	45,608	1,425	3.1%	56.3%
Marital status				
Married	252,101	32,064	12.7%	55.5%
Single	151,244	6,779	4.5%	55.4%
Divorced	48,042	4,192	8.7%	53.2%
Widowed	20,073	926	4.6%	52.7%
Unknown	8,183	165	2.0%	52.5%

Facility, department, and service comparisons

**Figure 5:**
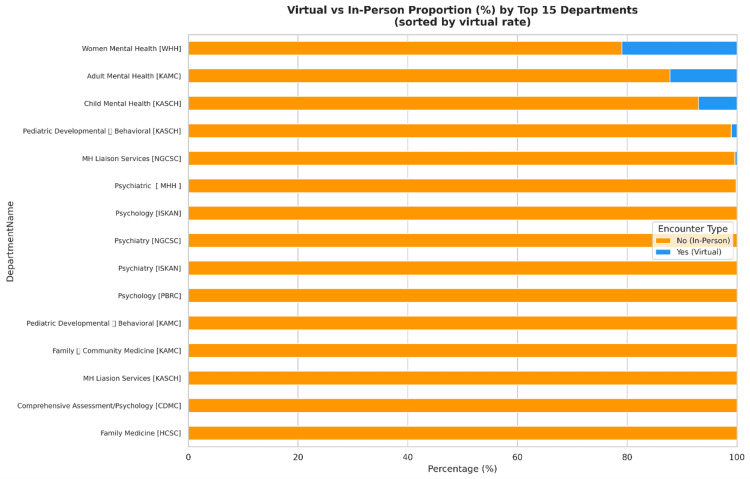
Virtual versus in-person proportion (%) by top 15 departments (sorted by descending virtual rate). Each stacked bar represents the percentage of appointments classified as virtual (blue) versus in-person (orange) within that department. Departments are ordered from the highest to the lowest virtual utilization rate. Substantial variation across departments reflects differences in clinical suitability and local operational practices for telehealth delivery. Only the 15 departments with the highest virtual rates are displayed; 29 unique departments were present in the full dataset (n = 479,643).

**Figure 6:**
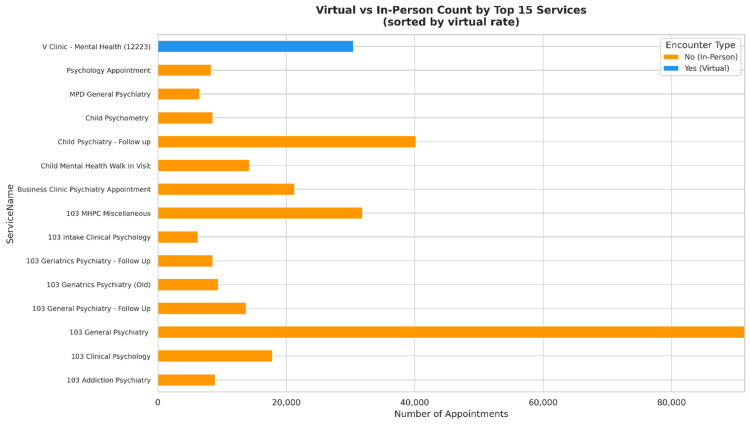
Virtual versus in-person proportion (%) by top 15 services (sorted by descending virtual rate). Each stacked bar shows the percentage of appointments within a service classified as virtual (blue) versus in-person (orange). Services are ordered from the highest to the lowest virtual utilization rate. Absolute appointment counts annotated above or within bars indicate service volume, illustrating that high virtual rates are not confined to low-volume services. Service-level variation parallels department-level patterns. Only the 15 services with the highest virtual rates are displayed; 199 unique services were present in the full dataset.

**Figure 7:**
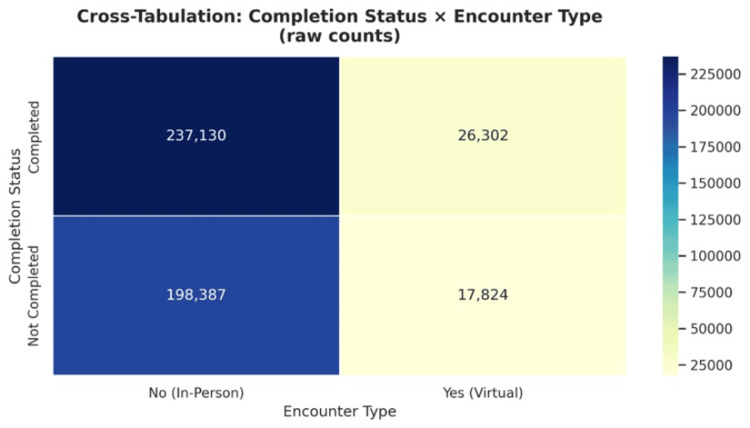
Cross-tabulation heatmap: completion status by encounter type. Cell values indicate the count of appointments within each combination of completion status (rows: Completed, Not Completed) and encounter type (columns: Virtual, In-Person). Color intensity reflects cell count magnitude, with darker shading indicating higher frequencies. Both encounter types show more completed than not-completed appointments; however, the virtual group has a proportionally higher share of completed appointments (59.6%) compared to the in-person group (54.4%), consistent with Table 6 and the logistic regression result (OR = 1.275, p < 0.001).

The dataset contains 12 named facilities, 29 departments, and 199 services. Figure 5 shows the virtual versus in-person comparison for the top 15 departments (sorted by virtual rate). Departments vary considerably in their telehealth adoption, reflecting heterogeneity in clinical suitability or operational practice. Figure 6 shows the virtual versus in-person proportion for the top 15 services (sorted by virtual rate). Service-level variation parallels department-level patterns, with some services showing high virtual proportions and others almost entirely in-person. Cross-tabulation by appointment type and encounter type shows that new patient appointments are almost exclusively in-person (1,181 virtual vs 128,588 in-person), while follow-up visits account for the largest share of virtual encounters (28,219 virtual vs 202,605 in-person). Figure 7 presents a cross-tabulation heatmap; both encounter types show more completed than not-completed appointments. The virtual group has proportionally more completed appointments.

Statistical testing results

Chi-square tests of independence were conducted between encounter type (yes [virtual] vs no [in-person]) and all categorical variables. Cramer's V is reported as the effect size. Table 8 presents a summary of all nine tests. The department name (top 10) comparison produced the largest effect size (V = 0.141), followed by pandemic period (V = 0.141), facility type (V = 0.102), and marital status (V = 0.086). The remaining comparisons had small effect sizes (V = 0.022-0.050), consistent with significance being driven partly by the large sample size (n = 479,643). The Mann-Whitney U test for age by encounter type yielded U = 1.15 x 10^11^, p < 0.001, rank-biserial r = -0.194 - a small-to-moderate effect, indicating that in-person patients tend to be younger. Non-normality was confirmed using the Shapiro-Wilk test (p < 0.001 in both groups).

**Table 8:** Summary of statistical test results (alpha = 0.05) Note: Nationality x encounter type (!): minimum expected cell = 0.09 (< 5) due to rare nationality groups, interpret with caution. H0, null hypothesis of independence or identical distributions; rbc, rank-biserial correlation; V, Cramer's V

Comparison	chi-sq / U	df	p-Value	Effect Size	Decision	n
Completion status x encounter type	430.46	1	<0.001	V = 0.030	Reject H0	479,643
Gender x encounter type	953.33	1	<0.001	V = 0.045	Reject H0	479,643
Nationality x encounter type (!)	231.87	52	<0.001	V = 0.022	Reject H0	479,643
Marital status x encounter type	3,508.03	4	<0.001	V = 0.086	Reject H0	479,643
Appointment type x encounter type	1,206.86	2	<0.001	V = 0.050	Reject H0	479,643
Facility type x encounter type	5,027.70	1	<0.001	V = 0.102	Reject H0	479,643
Pandemic period x encounter type	9,483.37	2	<0.001	V = 0.141	Reject H0	479,643
Department (top 10) x encounter type	9,432.81	9	< 0.001	V = 0.141	Reject H0	430,476
Age by encounter type (Mann-Whitney U test)	1.15e+11	—	<0.001	rbc = -0.194	Reject H0	479,639

Logistic regression: predictors of appointment completion

A binary logistic regression was fitted with Completed Flag (Completed = 1) as the outcome variable. Predictors included age, encounter type, gender, appointment type, facility type, and pandemic period, all available in the dataset. Dummy coding was applied with the following reference categories: No (In-Person), Female, First Visit, Hospital, Pre-Pandemic. The model was estimated on 479,639 records (four records were excluded for missing birthdate affecting age derivation).

Model fit was acceptable: observations = 479,639; McFadden's pseudo R² = 0.252; AIC (Akaike information criterion) = 493,951.94. Table 9 presents all coefficients. Virtual encounter type was a significant positive predictor (OR = 1.275; p < 0.001), indicating that virtual appointments were 27.5% more likely to be completed after adjusting for all other variables. The New Patient appointment type was the strongest negative predictor (OR = 0.036), consistent with its 40.3% completion rate. Gender (male vs female) was the only non-significant predictor (p = 0.639).

**Table 9:** Logistic regression: predictors of appointment completion Note: Reference categories were No (In-Person), Female, First Visit, Hospital, Pre-Pandemic, estimated by maximum likelihood using 200 iterations. AIC, Akaike information criterion; PHC, primary health care

Predictor (Reference)	Odds Ratio	95% CI	p-value	Interpretation
Age (per year, continuous)	0.997	0.996–0.997	<0.001	Slightly lower odds per year
Yes (virtual) vs no (in-person)	1.275	1.244–1.307	<0.001	Virtual: 27.5% higher odds
Male vs female	1.003	0.989–1.018	0.639	Not significant
Follow-up visit vs first visit	0.852	0.839–0.866	<0.001	Lower odds than first visit
New patient vs first visit	0.036	0.036–0.037	<0.001	Markedly lower odds
PHC vs hospital	1.111	1.084–1.138	<0.001	PHC: 11% higher odds
During pandemic vs pre-pandemic	1.497	1.461–1.534	<0.001	Higher during pandemic
Post-pandemic vs pre-pandemic	1.211	1.186–1.237	<0.001	Higher post-pandemic
n = 479,639 | McFadden’s pseudo R^2^ = 0.252 | AIC = 493,951.94	-	-	-	-

## Discussion

Main findings and interpretation

Across seven years, we analyzed 479,643 mental health appointments at King Abdulaziz Medical City. The study shows a shift from no virtual encounters before the pandemic to a sustained service afterward. Since 2021, virtual encounters have consistently made up 11% to 12% of annual encounters, suggesting telemental health has become an established service rather than a temporary emergency response. Our results also found that virtual encounters were more likely to be completed in comparison with in-person visits [7] and that the users were older on average. However, almost all new patients are seen in person, which could indicate that virtual mental health services are used for follow-up care rather than first contact.

After adjusted analysis, the virtual encounter type remained a positive predictor of completion after adjustment for age, gender, appointment type, facility type, and pandemic period, which is supported by the interpretation of the results. However, the effect size for completion was small, and thus the practical meaning is that the method of delivery matters, but it is only one part of appointment completion. Pandemic period and department type showed stronger associations than most demographic variables, suggesting that service organization and clinical context shape telemental health use more than patient characteristics alone.

Comparison with previous studies

Our findings are aligned with a recent study that showed that virtual appointments are more likely to be completed than in-person encounters [5]. In addition, they are also consistent with studies that have been conducted during the COVID-19 pandemic, which reported reduced no-show rates with increased satisfaction with telehealth [3,4]. The findings of our analysis are extending this pattern after the pandemic. Telemental health is still active with users rather than being discontinued after restrictions were lifted. This persistence supports the view that virtual care can serve as a stable, ongoing method of hybrid care delivery rather than a temporary, pandemic-only service [3,4].

The Saudi Arabian literature is supportive of these findings [13-15]. Local studies have reported positive clinician attitudes toward telemental health, patient acceptance, and good perceived quality [13-15]. Additionally, they identified operational barriers such as workflow variation and technical interruptions [7-9,13-15]. Our analysis supports those studies, which provided longer-term institutional data rather than just cross-sectional perception data alone. The expansion of virtual care services and digital health planning is supported by the sustained post-pandemic use that also fits the national digital health direction in Saudi Arabia [1,2,12].

Contribution to knowledge

By providing a seven-year, encounter-level overview of a Saudi Arabian tertiary hospital, this research project provides a unique perspective on how virtual mental health services are being used, especially after the pandemic. Much of the existing literature focuses on short pandemic use and perceptions, which represent a small period of time [10,17,18]. In this study, we combined utilization trends, completion outcomes, and patient characteristics in one dataset analysis. This way, we can possibly study why virtual mental health care expanded and understand how it was used and for whom it was most beneficial. This article provides a local view of operational evidence, which can support service planning, workforce allocation, and quality monitoring.

Strengths and limitations

With nearly half a million appointments included and no missing values in the original study variables, we can conclude that this project’s strength is the large sample size, which has not been produced in the Saudi Arabian literature before. This improves precision and reduces sampling error in the analysis. Additionally, we used complementary methods, including effect size and regression, which strengthened the interpretation beyond simple counts. A further strength is the long follow-up period, which allowed comparison of the pre-pandemic, pandemic, and post-pandemic patterns.

As some statistically significant findings had small effect sizes, they could be interpreted as operational associations rather than strong causal effects. The study has its own limitations, as it is a single-center retrospective analysis. The results were based on appointment completions, not symptomatic improvement or cost-effectiveness. Another limitation is that we could not study user satisfaction. We still need to cover important factors such as distance to care, digital access, and clinician preferences.

Implications for practice and future research

The findings support the continued use of virtual mental health as part of hybrid models, especially for follow-up appointments that could have an impact on access to care. We also need to look into new patient visits, which still have greater face-to-face input in many cases for mental health practices. In theory, this means that telehealth should be targeted toward clinical workflows where it works best rather than treated as a complete replacement for in-person care.

We need to focus more in future research on clinical diagnosis, symptoms, outcomes, patient satisfaction, and cost-effectiveness. We also need to look into multicenter Saudi Arabian studies that could potentially help determine whether these patterns are consistent across settings and in different places. An analysis of new patient visits should be investigated since they remain overwhelmingly in-person encounters, and we should study whether selected assessments could be safely shifted to virtual mental healthcare. Special work is also needed on equity, especially for different age groups, services, and access-to-care conditions.

## Conclusions

This study provided a seven-year institutional evaluation of telemental health utilization and patient engagement at King Abdulaziz Medical City, representing one of the first long-term analyses of its kind in Saudi Arabia. The findings showed that telemental health evolved from limited pre-pandemic use into a sustained component of routine mental healthcare delivery after 2021, indicating that virtual care became an integral service rather than a temporary response to COVID-19. Virtual appointments also demonstrated higher completion rates and lower no-show rates compared with in-person visits, suggesting stronger patient engagement. The study further showed that telemental health was used mainly for follow-up care rather than new patient assessments, supporting its role within a hybrid care model instead of replacing face-to-face services entirely. Future research should examine clinical outcomes, patient satisfaction, cost-effectiveness, and multicenter trends across Saudi Arabia and the Arabian Gulf region.
